# Supplementary material for: Effectiveness and safety of immunosuppressive regimens used as maintenance therapy in kidney transplantation: The CESIT study
Source: PLoS One. 2024 Jan 2;19(1):e0295205. doi: 10.1371/journal.pone.0295205 (PMC10760756; doi:10.1371/journal.pone.0295205)
Supplement: S1 Table — (DOCX) [file pone.0295205.s001.docx]

**S1 Table. Codes ICDIX-CM for infections**

| **Infections** | **ICDIX-CM codes** |
| --- | --- |
| Pneumonia and Influenza | 480, 481, 482, 483, 484, 485, 486, 487 |
| Urinary tract infections | 5990 |
| Tuberculosis and other mycobacteria | 010, 011, 012, 013, 014, 015, 016, 017, 018, 031 |
| Cytomegalovirus | 0785 |
| Herpes Virus | 052, 053, 054 |
| Pneumocysosis | 1363 |
| Cryptococcosis | 1175 |
