# Supplementary material for: Effectiveness and safety of immunosuppressive regimens used as maintenance therapy in kidney transplantation: The CESIT study
Source: PLoS One. 2024 Jan 2;19(1):e0295205. doi: 10.1371/journal.pone.0295205 (PMC10760756; doi:10.1371/journal.pone.0295205)
Supplement: Attachment — Submitted filename: Answers_Reviewers_11092023.docx [file pone.0295205.s002.docx]

**Reviewer #1**: The manuscript by Bellini and colleagues reports the results of an observational (registry) study on the use of CNI based maintenance therapies in kidney transplant recipients, using propensity score matching.
Their results confirm in a large cohort included over a 10 years period, important findings such as the superiority of tacrolimus as compared to CsA for graft outcomes and lower risk of diabetes as well as higher risk of dyslipidemia as defined by statin use for the combination tac+mTORi as compared to tac+MMF. The abstract mentions a potential protective effect of mTORi+Tac as compared to MMF+Tac that do not reach statistical significance and is not supported by RCTs (eg Transform).
Sensitivity analysis are performed accounting for meaningful clinical variables.

Major:
The nature of registry data significantly limits granularity of the analysis, being the most important outcome a combination of rejection/graft loss.
Can the author clarify the definition of this variable? Is rejection reliably recorded (Biopsy?discharge diagnosis? Receiving rescue treatment?) in this registry? In case not, only graft loss might be a more accurate outcome since it depends on clear events such as starting dialysis or retrasplantation. If, on the other hand, rejection IS reliably recorded, splitting the outcome in two might be interesting since graft loss is of course not related to alloimmune events in a large percentage of cases.

*We thank the reviewer for the comment. Information on transplant rejection was detected by the Transplant Information System an infrastructure for the management of data related to the activity of the National Transplant Network, established and regulated by Italian Laws (n. 91 of April 1, 1999). The SIT collecting data on donation, allocation and transplantation of organ including transplanted organ quality, through SIT it is possible to ensure transparency and traceability of the donation, retrieval and transplantation processes. Specifically, data on organ rejection is detected directly by clinicians following a finding of impairment of transplanted organ function due to histologically documented immunological causes. Furthermore, data quality qudits and checks are frequently performed. We added these details in the method section.*

Minor:
Were recipients of other solid organ transplants excluded from the analysis?

*Yes, we excluded multi-organ transplant recipients. We added details on this point in the method section and in the flow-chart (Figure 1). More details about the selection of the cohort can be found in the previous paper:*

*Belleudi V, Rosa AC, Finocchietti M, Poggi FR, Marino ML, Massari M,
Spila Alegiani S, Masiero L, Ricci A, Bedeschi G, Puoti F, Cardillo M, Pierobon S, Nordio M, Ferroni E, Zanforlini M, Piccolo G, Leone O, Ledda S, Carta P, Garau D, Lucenteforte E, Davoli M and Addis A, CESIT Study Group (2022), An Italian multicentre distributed data research network to study the use, effectiveness, and safety of immunosuppressive drugs in transplant patients: Framework and perspectives of the CESIT project. Front. Pharmacol. 13:959267.*

It would be useful to report mean or median follow-up time of the cohort.

*We appreciate the reviewer's suggestion, we added this information in the result section.*

In line 119 page 5 ,HLA-DL--> DR?

*You are right we have corrected the typo*

Is the outcome “diabetes” restricted to post transplant? Please define in methods.

*As reported in the methods section “for each outcome, only patients who were at risk of developing the outcome for the first time were considered” (Page 6). To clarify this point, we modified the manuscript by referring to new-onset diabetes.*

Does Table 2 show Hazard ratios? the note has maybe some incongruence with the table or are SMD shown here?

*Yes, Table 2 shows HR. To clarify this point, we added description in the table.*

**Reviewer #2:** I thoroughly reviewed the paper and found it to be both interesting and pertinent. Despite the abundance of existing literature on the topic, it's crucial to acknowledge potential variations in data across different locations. Assessing the effectiveness and safety of various immunosuppressive regimens used in kidney transplantation adds significant value to the overall comprehension of the subject.

My comments and questions are as follows:
1) Page 2 line 39 - Abstract: The supposed benefit of mTORi usage, as it was not statistically significant, should not be stated in the abstract as a certainty. It could be described as non-inferiority instead.

*We agree with you. We modified the abstract following your suggestion.*

2) Methods: Elaborate further on the specific parameters used to classify infections as severe, and provide a detailed description of the methodology employed to gather the necessary data for conducting the effectiveness analysis in relation to these severe infections.

*The infection-related outcome was based on only severe infections, which were defined as those resulting in hospitalization. We have included in S1 Table the ICD-IXCM codes selected for this outcome and in References section some papers on the basis of which we made the choice of codes.*

3) Methods: why wasn't the type of donor considered in PS-matching?

*We apologize to you, we forgot to indicate this in the list of variables considered, although we had included it in PS-matching (as shown in Figure 2 A/B). We corrected the method section.*

4) Methods: Elaborate further on the specific parameters used to classify transplant hospital length as standard or prolonged

*Prolonged hospitalization was defined as a length of stay equal to or greater than the 75th percentile of length of stay of all participants*

5) Page 5 line 119 - Correct spelling : length of transplant

*You are right we have corrected the typo*

6) Page 7 line 164 - Correct spelling : HLA- DR

*You are right we have corrected the typo*

7) Missing Explanation for Certain Adjustments: While the article adjusts for infections, malignancies, and delayed graft function (DGF), it might be helpful to provide a brief rationale for these adjustments and their relevance in the context of the study.

*Thank you for your suggestion, we have modified the text and the bibliography by inserting a brief rationale regarding adjustments and by adding some previous work on which we had based our choice of adjustments*

8) Graphics - enhance the resolution and select colors more effectively to improve visual clarity.

*We modified resolution and colors to improve the graphics. We are available to consider further changes with the editor if the paper will be accepted for publication.*

9) Results: Explore in the text information obtained from graphs 5A-F.

*We added a commentary paragraph on graphs in the result section.*

10) Page 15 line - Correct spelling: prednisone-use

*You are right we have corrected the typo*

11) The study fails to address a crucial aspect, namely medication dosages. Since the study relies on medication dispensation data, a substantial bias arises concerning the dosages of calcineurin inhibitors and mTORi. Considering the decade-long selection and monitoring period, it's reasonable to assume that patients in the tacrolimus group encompassed both low and high dosage users. This differentiation could significantly impact outcomes like the incidence of NODAT. Furthermore, distinguishing dosage variations in the tacrolimus mTORi/MPS -associated groups could provide more accurate insights into the actual extent of the potential benefits of mTORi usage. I recommend a deeper exploration of this bias within the article's limitations section. While the study provides valuable insights into immunosuppressive regimens in kidney transplantation, it has some notable gaps. The lack of details about medication dosages, potential confounding factors, and inherent biases in retrospective data are concerning. Additionally, the interpretation of results and the discussion could have more critically addressed limitations and potential clinical implications of the findings. This could strengthen the validity and clinical applicability of the study's conclusions.

*Thank you for your comment, this is indeed an important point of the limitations of the study and an important element to be explored with future investigations, we have included a sentence in the limitations section to emphasise the lack of consideration of dose and the possible bias concerning this.*
