# Supplementary material for: Effectiveness and safety of immunosuppressive regimens used as maintenance therapy in kidney transplantation: The CESIT study
Source: PLoS One. 2024 Jan 2;19(1):e0295205. doi: 10.1371/journal.pone.0295205 (PMC10760756; doi:10.1371/journal.pone.0295205)
Supplement: Attachment — Submitted filename: Answers_Reviewers.docx [file pone.0295205.s004.docx]

**Reviewer #3**: The manuscript evaluated the effectiveness and safety by comparison between cyclosporine and tacrolimus-based therapies, Tacrolimus + mTORi and Tacrolimus + MMF in kidney transplant recipients and reported the results of real-world data on the use of CNI based maintenance therapies in kidney transplant recipients.
1.It would be very important to report baseline renal function ( serum creatinine ,GFR or eGFR ) and urine protein of transplant kidney.

*Thank you for the suggestion since the work in based on administrative data we had limited access to clinical information about our cohort. Data regarding baseline renal function were not available within the SIT; we had some data on renal function after transplantation (GFR). However, we chose not to add renal function as an effectiveness outcome because the variable resulted in a high rate of missing values (exceeding 80% for some of the years considered). We are aware that this is an important limit of the study and we have rephrased the discussion, emphasizing the importance of investigating this aspect in future studies.*

2.The outcome considered in the manuscript were mortality and transplant reject/graft failure for effectiveness analysis, and incidence of severe infections, cancer, diabetes and statin use for safety analysis. The outcome should consider renal function progression. ≥50% decline in eGFR should be added to the primary outcome for effectiveness analysis.

*As previously mentioned, the clinical information about renal function is not available. The study is observational, based on administrative claims and CNT data, this limitation is addressed in the discussion.*

**Reviewer #4**: Dear authors,
I think the revised form of the manuscript is quite improved. I will hava e few additional comments.
The use of mTOR inhibitors as part of initial maintenance therapy is usually limited by early posttransplant complications (delayed allograft function, poor wound healing, and an increased incidence of lymphoceles) associated with these agents. Did you have any data about this? Were mTOR inhibitors used in these patients as a part of initial maintenance therapy?

*No, we don’t have this data. We have outlined this limitation in the discussion section. However, our study revealed that in some medical centers, mTORi are prescribed in 60% of cases, and it is unlikely that all of this usage can be attributed to these complications.*

Were the ones under the treatment of azathioprine excluded?

*Yes, we excluded these patients. We added this information in the method section.*

Lack of data about induction treatment is an important limitation. Data whether target levels of CNI’s were reached or not was also lacking.

*No, the nature of administrative data precludes the acquisition of detailed clinical patient information. Nevertheless, we believe that this work can enhance the understanding of maintenance immunosuppression and its use. As reported in the discussion section, the result observed in our unselected population are similar to those seen in trials. Despite these limitations in data availability, we do not believe that these factors have introduced distortions in our study.*

Did you know BK virus infection prevalance in these different groups?

*The ICD-9CM come for tracking BK virus infection is 079.89, which corresponds to “Other specified viral infection”. Since it is not an exclusive and specific code, we have decided not to use it for tracking infections. However, this aspect is certainly interesting and we will consider it for future project developments.*

P64 line 99 LAR should be explained. (legally acceptable representative)

*Thank you, we have explained the acronym.*

Table 1 “sovrappeso” should be corrected.

*We have translated the word.*

**Reviewer #5**: *This is an interesting study that tried to compare the outcomes of kidney transplant patients whether they were on tacrolimus or cyclosporine and whether they were on MMF or mTORi. They used the PS matching to make these comparisons on a real-world cohort.*
Although the PS matching is the best way to reduce any confounding bias, the authors lost almost half of the sample because they could not match them. When we look at Fig 2A we can see that the differences between those matched or not for the comparison of TACROLIMUS to CYCLOSPORINE do not include cancer, comorbidities, metabolic parameters, etc..however, in Fig2B, the differences are more important especially when it comes to cancer that is one of the indicatiosn to switch to mTORi. Therefore, authors need to align their conclusions with this important limitation of the PS matching selection.
My other comment is that this manuscript should be revised by a native English speaker (some sentences are really hard to read).

*We lost half of the sample, but this reduction was mainly due to the fact that there were only 787 CsA users; hence, the patients for whom no match was found were only 64 (787-723). The study aimed to analyze the association between therapies and outcomes and we chose to use statistical methods that allowed for internally valid results, even though this may result in a reduced external validity.*

*We added a reference in the text where Rubin DB and colleagues demonstrated that propensity score matching is the best way to eliminate differences between groups (42).*

- *Rosenbaum PR, Rubin DB. The central role of the propensity score in observational studies for causal effects. Biometrika. 1983;70:41–55.*
- *Rubin DB. Using multivariate matched sampling and regression adjustment to control bias in observational studies. J Am Stat Assoc. 1979;74:318–328. [*[*Google Scholar*](https://scholar.google.com/scholar_lookup?journal=J+Am+Stat+Assoc&title=Using+multivariate+matched+sampling+and+regression+adjustment+to+control+bias+in+observational+studies&author=DB+Rubin&volume=74&publication_year=1979&pages=318-328&)*]*
- *Rubin DB, Thomas N. Matching using estimated propensity scores: relating theory to practice. Biometrics. 1996;52:249–264. [*[*PubMed*](https://pubmed.ncbi.nlm.nih.gov/8934595)*] [*[*Google Scholar*](https://scholar.google.com/scholar_lookup?journal=Biometrics&title=Matching+using+estimated+propensity+scores:+relating+theory+to+practice&author=DB+Rubin&author=N+Thomas&volume=52&publication_year=1996&pages=249-264&pmid=8934595&)*]*

*Figures 2a and 2b demonstrate that after matching all the characteristics between the groups are balanced.*

**Reviewer #6**: Really interesting stuff.

Keywords: Please uniform. Either all in lowercase, or all in uppercase. Choose and be consistent.

Is 'raw data' available?
This is very important, the verifiability and repeatability of the research. It is unclear whether or not.

As reported in the manuscript “The data that support the findings of this study are available from the Italian regions participating to CESIT study but restrictions apply to the availability of these data, which were used under license (as by third-party sources) for the current study, and so are not publicly available. However, data are available with permission of Italian regions, which are the data owner. The non-author contact information to which data requests may be sent is: [project.cesit@gmail.com](mailto:project.cesit@gmail.com).”

Specific request will be evaluated.

I agree that the outcome of rejection must be histologically documented.

*Ok. We reported this information in the method section.*

P2 L38: Please, write 'risk of rejection/' instead of 'reject/'.

*Ok.*
P4 L77: Avoid abbreviations at the very beginning of the sentence.
etc.

*Ok.*

Go through the text a few more times, there are still some lexical and grammatical errors.

*Ok.*

*We have corrected the errors.*

P4 L81: There is still the question of correction of IS therapy in the event of malignancy, conversion to mTOR, etc., where even some of our national transplant experts cannot give an unequivocal answer or conclusion as to what to do.
As you said - guidelines are one thing, life is another.

*This is an interesting question, but the answer of this concern was out of scope of our work.*

**Reviewer #7**: *In the current manuscript, Bellini et al. reveal the effectiveness and safety profile of different immunosuppressive regimens in kidney transplantation. A large amount of multicenter data was collected and statistical analysis was performed. Their results indicated that tacrolimus-based immunosuppressive therapy appeared to be superior to cyclosporine in reducing rejection and severe infections. Besides, the combination of tacrolimus and mTORi may represent a valid alternative to the association with mycophenolate. These results are very important to improve graft survival and reduce acute rejection.*

1.Mortality and transplant reject/graft failure were defined as outcomes of this study. Is it also possible to include renal function like eGFR as the outcomes? To use renal function as an indicator of effectiveness could help clinicians to evaluate the prognosis. I recommend a deeper exploration of this outcome if it is possible.

*Thank you for your suggestion. Even though we agree that renal function would be interesting to investigate in our cohort, we have not included it as one of the outcomes because the study is based on administrative data and we had limited access to information on renal function. The variable related to GFR resulted in a high rate of missing values (exceeding 80% for some of the years considered). We emphasized this aspect in the discussion section.*

2. Please unify the wording, such as tumor and cancer.

*Thank you for the suggestion, we have replaced the word tumor with cancer in the text.*

3.The study relies on medication dispensation data where dosage is lacking. However, medication dosage is a crucial factor to assess the adverse outcome. I noticed hospital information system and co-payment exemption registry. Is it possible to acquire the dosage information by fee items or claims?

*Dosage in administrative data can be obtained using “Defined Daily Dose” (DDD), which is the assumed average maintenance dose per day for a drug used for its main indication in adults. However, it is likely that in the context of transplant the therapeutic doses used in clinical practice will be different from the DDD and will depend on individual patient characteristics. This aspect was already mentioned in the limits, we have further clarified it in the discussion.*

4. Method - Given the new onset of diabetes and statin use are ones of the outcome in the study, it’s inappropriate to have the history of diabetes and statin use at baseline as covariates. Please excluded patients with these two factors from the beginning.

*We had already excluded patients with history of diabetes and statin use from the analysis; we have rephrased the sentence in the methods section for better clarity (L140-142)*

5. Result - Table 1 tells several indicators’ SMD value of comparison pairs between TAC+MMF and TAC+mTORi ≥0.1. The indicators should be included as confounders in Cox models to assess the independent effect of exposures.

*Ok. As reported in the method we included in the model adjustment for propensity score value*

6. Result - The 112 (20%) patients of TAC+mTORi group were excluded according to the data after propensity score matching, which may result in sampling bias. Please use one to more matching or provide the outcome of the excluded data.

*The study’s objective was to examine the relationship between therapies and the outcomes consideres; we opted for statistical methods that prioritize internal validity, albeit potentially reducing external validity*.

*Rubin DB and colleagues demonstrated that propensity score matching is the best way to eliminate differences between groups (42).*

- *Rosenbaum PR, Rubin DB. The central role of the propensity score in observational studies for causal effects. Biometrika. 1983;70:41–55.*
- *Rubin DB. Using multivariate matched sampling and regression adjustment to control bias in observational studies. J Am Stat Assoc. 1979;74:318–328. [*[*Google Scholar*](https://scholar.google.com/scholar_lookup?journal=J+Am+Stat+Assoc&title=Using+multivariate+matched+sampling+and+regression+adjustment+to+control+bias+in+observational+studies&author=DB+Rubin&volume=74&publication_year=1979&pages=318-328&)*]*
- *Rubin DB, Thomas N. Matching using estimated propensity scores: relating theory to practice. Biometrics. 1996;52:249–264. [*[*PubMed*](https://pubmed.ncbi.nlm.nih.gov/8934595)*] [*[*Google Scholar*](https://scholar.google.com/scholar_lookup?journal=Biometrics&title=Matching+using+estimated+propensity+scores:+relating+theory+to+practice&author=DB+Rubin&author=N+Thomas&volume=52&publication_year=1996&pages=249-264&pmid=8934595&)*]*

*We added a reference in the paper.*

7. Graphics - Figure 2A and 2B were seen unclear here. Please provide clearer figures.

*OK. We modified the figures.*

**Reviewer #8:** (No Response)
